# Hypoxia-inducible MiR-182 promotes angiogenesis by targeting RASA1 in hepatocellular carcinoma

**DOI:** 10.1186/s13046-015-0182-1

**Published:** 2015-06-28

**Authors:** Chengli Du, Xiaoyu Weng, Wendi Hu, Zhen Lv, Heng Xiao, Chaofeng Ding, Owusu-anash K. Gyabaah, Haiyang Xie, Lin Zhou, Jian Wu, Shusen Zheng

**Affiliations:** Department of Hepatobiliary Surgery, The First Affiliated Hospital, Zhejiang University School of Medicine, Hangzhou, 310003 China; Collaborative Innovation Center for Diagnosis and Treatment of Infectious Diseases, The First Affiliated Hospital, Zhejiang University School of Medicine, Hangzhou, 310003 China

**Keywords:** HCC, miR-182, Angiogenesis, Hypoxia, RASA1

## Abstract

**Background:**

Hypoxia is a common feature of solid tumors, including HCC. And hypoxia has been reported to play an important role in HCC progression. However, the potential mechanism of miRNAs in hypoxia mediating HCC progression still remains unclear.

**Methods:**

The HCC cells were cultured in the atmosphere of 1 % oxygen to induce hypoxia. The microRNA microarray was employed to search for the hypoxia-inducible miRNAs. RT-PCR, western blot and immunohistochemistry were used to detect the RNA and protein levels. HUVEC were applied to explore the angiogenesis level.

**Results:**

We found that miR-182 was upregulated in the hypoxia-based microarray. We then revealed that miR-182 was also significantly increased in the HCC tissues compared to the corresponding normal tissues. In vitro capilliary tube formation assays showed that the miR-182 promoted angiogenesis. RASA1 was demonstrated as the direct target of miR-182. In addition, the suppression of RASA1 phenocopied the pro-angiogenesis effects of miR-182. Besides, RASA1 was also decreased in the hypoxia HCC cells while the inhibition of miR-182 partially restored the level of RASA1.

**Conclusions:**

Our data showed that hypoxia regulated the expression of miR-182 and RASA1 to promote HCC angiogenesis.

**Electronic supplementary material:**

The online version of this article (doi:10.1186/s13046-015-0182-1) contains supplementary material, which is available to authorized users.

## Introduction

Hepatocellular carcinoma (HCC) is one of the most common cancers, which lead to the third leading cause of death from cancer worldwide [1]. HCC shares the common character of tissue hypoxia with other solid tumors, due to the quick growth of tumor and relatively low density of microvessels. Upon hypoxic environment, HCC cells will subsequently lead to elevated angiogenesis and tumor metastasis, which is resistant to chemotherapy and cause the poor survival of HCC patients [2–4]. So investigation of hypoxia-induced molecules will help to develop new tools for the treatment of HCC.

MicroRNAs (miRNAs) are a class of highly conserved short RNAs of 21–23 nucleotides that negatively regulate protein expression via binding to the 3’untranslated region (3’UTR) of their targeted mRNAs [5, 6]. MiRNAs have been reported to play an important role in tumor progression, including apoptosis, proliferation, angiogenesis and metastasis [7–9]. To date, several miRNAs are identified to be regulated under hypoxic conditions and are recognized as hypoxic responsive miRNAs, which contribute to the tumor progression. For example miR-210, which is overexpressed in HCC, has been reported to be induced in hypoxic HCC cells and promote the metastasis of HCC by targeting VMP1 [10]. In addition, Lan J et al. reported that miR-497 is increased in hypoxic glioma cells and decreases their sensitivity to TMZ by inhibiting apoptosis [11]. MiR-182 has been reported to act as a tumor promoter in several cancer types [12, 13]. A previous study has demonstrated that miR-182 promotes metastasis in HCC [14]. However, the relationship between miR-182 and hypoxia still remains unclear.

In this study, we identified miR-182 as the hypoxia responsive miRNA by microRNA microarray analysis. Then we found that miR-182 was up-regulated in HCC tissues. In addition, we demonstrated that miR-182 promoted angiogenesis of HCC by targeting RASA1. Our results indicate that miR-182 promotes the adaptation of HCC under hypoxic conditions and might acts as a therapeutic target for HCC.

## Materials and methods

### Patients and tissue specimens

HCC tissues and their corresponding normal liver tissues were obtained from 36 HCC patients who underwent resection of HCC at the First Affiliated Hospital Zhejiang University School of Medicine, China. All tissue samples were freshly frozen by liquid nitrogen and stored at - 80 °C. All the specimens were obtained with informed consent and approved by the Ethics Committee of Zhejiang University.

### Cell lines and culture

Liver cancer cell lines SK-HEP-1 and HCC-LM3 cells were purchased from the Shanghai Institute of Cell Biology (Shanghai, China). The two cell lines were maintained in the Dulbecco’s modified Eagle medium with 10 % fetal bovine serum and in the atmosphere at 37 °C containing 5 % CO2. To induce hypoxia, cells were cultured in the atmosphere of 1 % O2, 5 % CO2 and 94 % N2.

### Preparation of tumor cell-conditioned medium (TCM)

HCC cells were transfected for 48 h and then the supernatant medium was replaced by serum free medium. After incubation for another 24 h, the medium was collected following by centrifuging for 10 min at 5000 g and stored at −80 °C until used.

### Human umbilical vein endothelial cells (HUVECs) and capillary tube formation assay

The HUVECs were obtained from Allcells (Shanghai, China allcells.biomart.cn) and maintained with Serum Free Medium for Endothelial Cells (SFM, Invitrogen) supplemented with 20 % FBS, 0.1 mg/mL of heparin and 0.03 mg/mL of endothelial cell growth supplement. Primary HUVECs were used at passages 2–7 in all experiments. For capillary tube formation assay, HUVECs were transferred to matrigel-coated 96-well plates by 75 % TCM at the density of 2 × 10^4^/well. After incubation for 6 h, capillary-like structures of HUVECs were photographed under an inverted microscope. The branch points of the formed tubes, which represent the degree of angiogenesis in vitro, were scanned and quantitated at the 100× magnification.

### RNA oligoribonucleotides and transfection

MiR-182 mimics (MSY0000259), inhibitors (MIN0000259), si-RASA1 (SI00045360) and AllStars negative control siRNA (SI03650318) were synthesized by Qiagen (Germany). The transfection was conducted using lipofectamine 2000 (Invitrogen) according to manufacturer’s construction.

### Luciferase reporter assay

SK-HEP-1 and HCC-LM3 cells were seeded in 24-well plates and then the cells were co-transfected with miR-182, anti-miR-182 or control and 100 ng either wild-type or mutant-type 3’UTR of RASA1 firefly luciferase reporter plasmid. After incubation for 48 h, firefly and renilla luciferase activities were measured by a dual-luciferase reporter assay (promega, E2920).

### RT-PCR, western blot and immunohistochemistry (IHC)

The regents used and the detailed procedures of RT-PCR, western blot and IHC were performed as before [7]. For RT-PCR, GAPDH and U6 were used as the internal control. The primers of GAPDH, RASA1 were listed in Table 1 and the primers of U6 (MS00033740) and miR-182 (MS00008855) were bought from Qiagen. For western blot, β-actin (A5441, Sigma-Aldrich, 1:2000) was used as the internal control. Other primary antibodies used were Hif-1a (ab113642, abcam, 1:1000), RASA1 (ab40677, abcam, 1:1000).

**Table 1:** Primer sequences for RT-PCR

Name	Sense strand	Anti-sense strand
GAPDH	GTCTCCTCTGACTTCAACAGCG	ACCACCCTGTTGCTGTAGCCAA
RASA1	GGGACATCCAATAAACGCCTTCG	TTTGCTACTTGGACACTATTCAGG

For evaluation of staining score of RASA1 (ab40677, abcam, 1:50), we applied modified Histo-score (H-score) method, which in brief was a semi-quantitative assessment of both fraction of positive cells (0–1) and intensity of staining (no 0, weak 1, moderate 2, or strong 3). The H-scores ranged from 0 to 3, which were obtained by multiplying the fraction and intensity scores.

### MiRNA microarray analysis

Agilent oligonucleotide technology was applied to profile the differentially expressed miRNAs between normoxia and hypoxia (8 h) groups. In brief, the RNA was isolated by mirVanaTM miRNA Isolation Kit (Cat#AM1560, Ambion, Austin, TX, US) and followed by labeling and hybridization with miRNA Complete Labeling and Hyb Kit (Cat#5190-0456, Agilent technologies, Santa Clara, CA, US). The microarray used for hybridization was Agilent human miRNA (8*60 K) V18.0. Then slides were scanned by Agilent Microarray Scanner (Cat#G2565BA, Agilent technologies, Santa Clara, CA, US) and Feature Extraction software 10.7 (Agilent technologies, Santa Clara, CA, US) with default settings. Raw data were normalized by Quantile algorithm, Gene Spring Software 11.0.

The microarray data was submitted to GEO database and the GEO accession number was GSE68593.

### Statistical analysis

The results are presented as the mean ± standard error of the mean (SEM). The data of miR-182 and RASA1 expression between groups were subjected to the Student *t* test (two-tailed). Mann–Whitney test was used to compare the difference between two groups. And for three groups, One-way ANOVA followed by Tukeys post-hoc test was applied. Fisher exact test was perform to examine the relationship between miR-182 expression and clinicopathological characteristics. Statistical analysis was performed with SPSS 15.0 and GraphPad Prism 5.0. *P* < 0.05 was considered statistically significant.

## Results

### Hypoxia induces the level of miR-182 in HCC cells

**Fig. 1:**
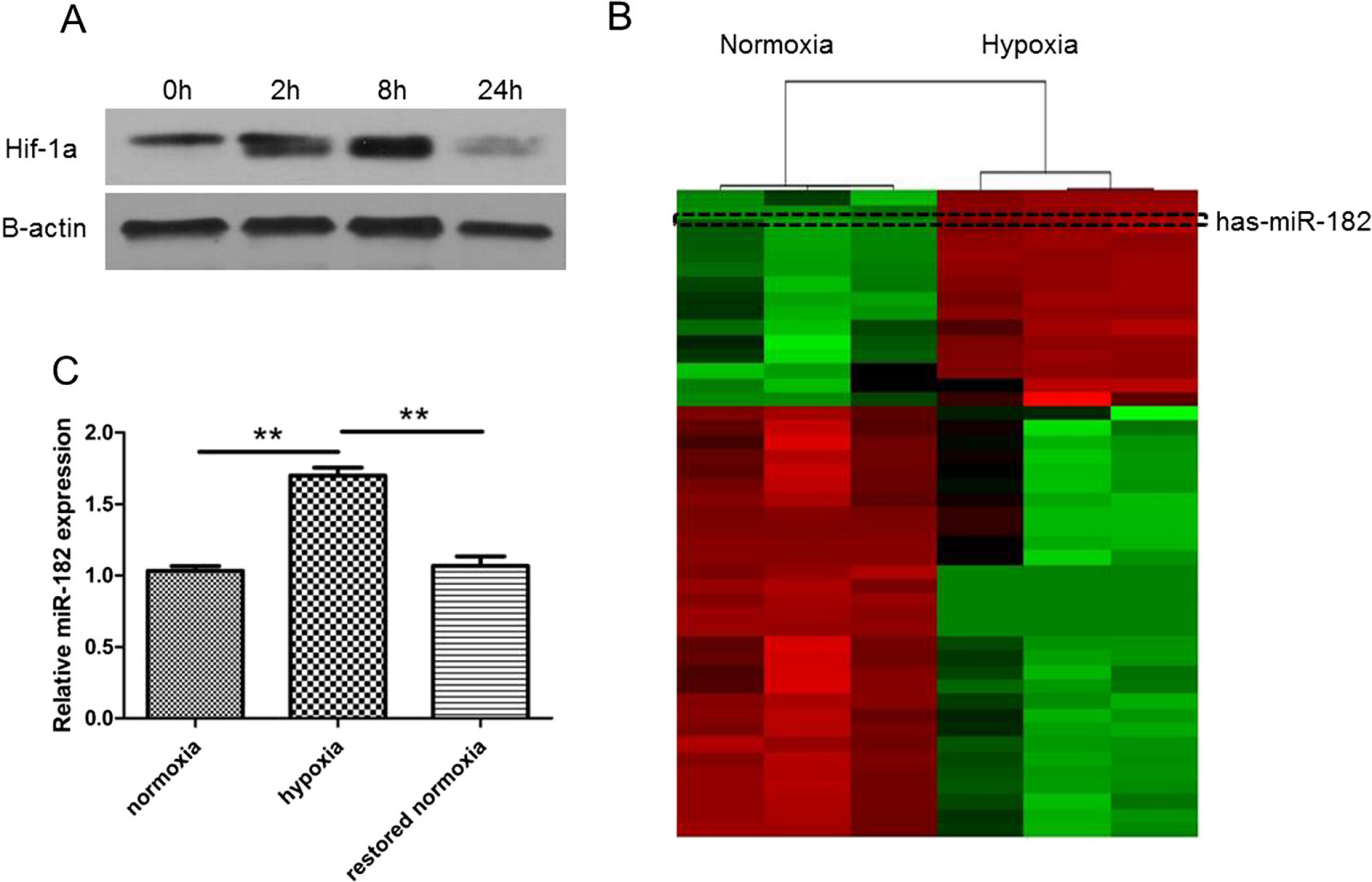
The expression of miR-182 was enhanced in the hypoxia HCC cells. **a** The level of hif-1a in SK-HEP-1 under different hypoxia exposure time (0 h, 2 h, 8 h, 24 h). **b** We applied the normoxia SK-HEP-1 cells and hypoxia SK-HEP-1 cells (8 h) for microRNA microarray. The unsupervised hierarchical clustering analysis showed a significant increase of miR-182 in the hypoxia group. **c** HCC cells were restored to normoxia after applied to normoxia for 8 h. The relative level of miR-182 was detected by RT-PCR. The experiments were performed in three independent times. (**P < 0.01)

Firstly, we applied SK-HEP-1 cells under hypoxia conditions for several time points. We then examined the hypoxia marker, hypoxia-inducible factor 1 alpha (Hif-1a), in each group. Western blot results showed that the Hif-1a level was significantly increased in the 2 h and 8 h time points, and decreased in the 24 h time point (Fig. 1a). To search for the hypoxia-inducible miRNAs, we performed microRNA microarray scanning of SK-HEP-1 cells subject to normoxia and hypoxia for 8 h. Among all the miRNAs profiled, we identified that miR-182 was the highly induced microRNA in the hypoxia group by setting the fold change > 2 and p value < 0.01 as the cut-off point (Fig. 1b).

To validate the microarray data, we then performed RT-PCR of miR-182 in SK-HEP-1 cells under normoxic and hypoxic conditions. We found that the expression of miR-182 was significantly increased under hypoxia (Fig. 1c). In addition, the miR-182 level was decreased after the HCC cells were restored in the normoxic condition for 24 h (Fig. 1c). Therefore, the data suggests that hypoxia could increase the expression of miR-182.

### MiR-182 is significantly increased in HCC tissues and promotes angiogenesis in HCC

**Fig. 2:**
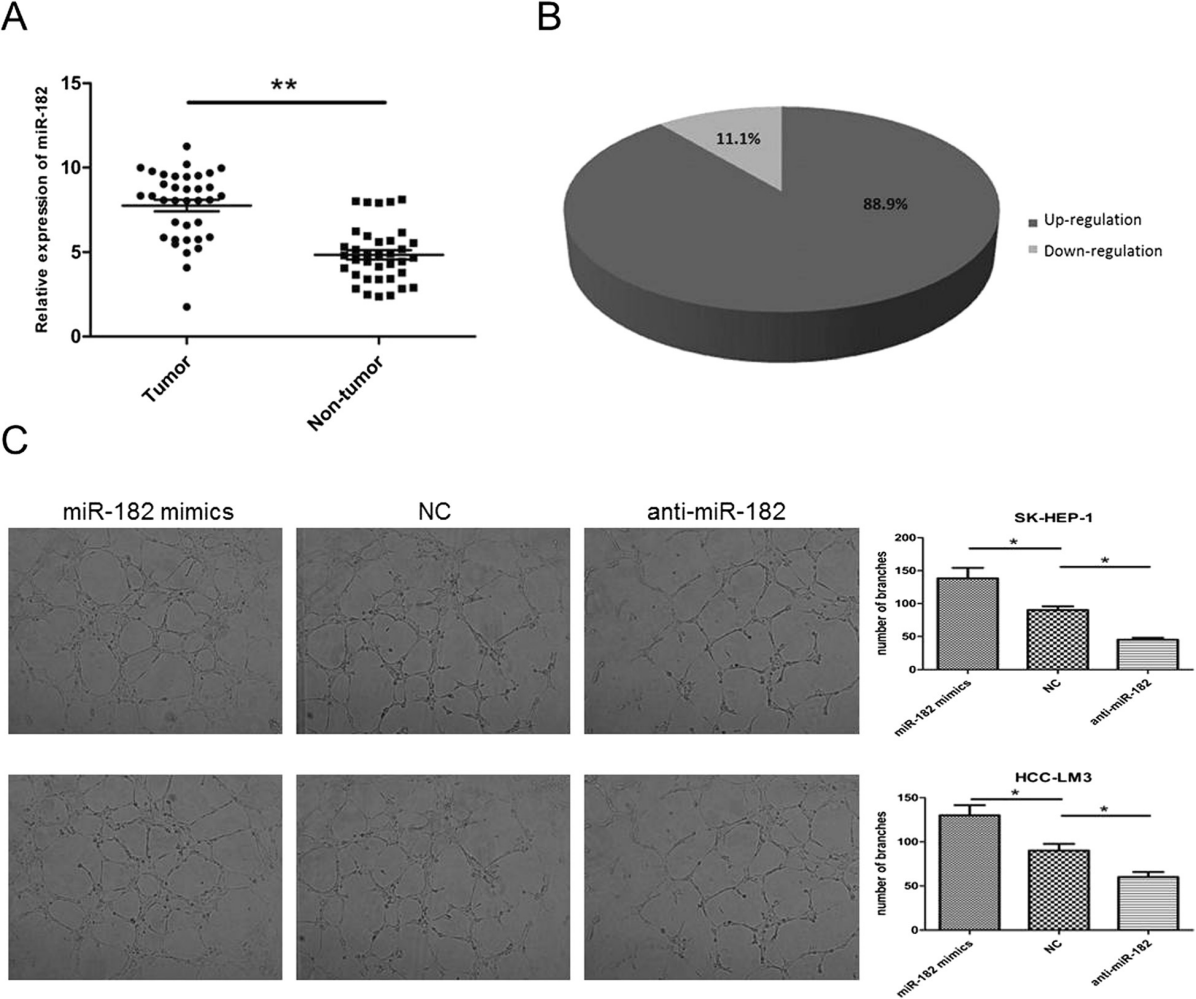
The level of miR-182 was increased in HCC tissues and promotes angiogenesis. **a** The level of MiR-182 was examined by RT-PCR in 36 pairs of HCC tissues and their corresponding normal tissues. **b** The percent of up-regulation of miR-182 in HCC was shown. **c** Capillary tube formation assays of HUVECs were performed by using 75 % TCM derived from SK-HEP-1 and HCC-LM3 cells treated with miR-182 mimics or NC or anti-miR-182. The relative images were shown at the 100× magnification and the results are representative of three independent experiments. (*P < 0.05, **P < 0.01)

We then explored the expression of miR-182 in 36 pairs of HCC tissues. Consistent with a previous study [14], the level of miR-182 was significantly increased in HCC tumor tissues compared to the corresponding normal tissues (Fig. 2a, b). We then examined the clinicopathologic significance of miR-182 in HCC by choosing the median of miR-182 as the cut-off point to separate the low-miR-182 group and the high-miR-182 group (Table 2). We found that the upregulation of miR-182 was more frequently observed in the patients with big tumor size (*P* = 0.018) and portal vein invasion (*P* = 0.027). However no significant correlation was found between miR-182 and other clinicopathologic data, including age, gender tumor number, TNM staging, grade and AFP level.

**Table 2:** The relationship between miR-182 expression and clinicopathological characteristics in human HCC patients

Variables	All patients	miR-182 expression		*P* value
	(n = 36)	low^a^	high^a^	
Age (years)				
≤55	12	7	5	0.725
>55	24	11	13	
Gender				
Male	28	13	15	0.691
Female	8	5	3	
Size of tumor (cm)				
≤5	16	12	4	0.018*
>5	20	6	14	
Number of tumor				
Single	20	12	9	0.5
Multiple	16	6	9	
Portal vein invasion				
Negative	25	16	9	0.027*
Positive	11	2	9	
TNM staging				
I, II	21	13	8	0.176
III	15	5	10	
Grade				
Well + Moderate	27	15	12	0.09
Poor	9	3	6	
AFP (ng/ml)				
≤400	10	6	4	0.711
>400	26	12	14	

For analysis of correlation between miR-182 expression and clinical features, fisher exact tests were used. Results were considered statistically significant at *P* <0 .05 *

^a^The median expression level was used as the cut-off. Low expression of miR-182 in 18 patients was classified as values below the 50th percentile. High miR-182 expression in 18 patients was classified as values above the 50th percentile

As hypoxia is an important factor to induce angiogenesis [15, 16], we predicted that hypoxic-induced miR-182 could also increase the angiogenesis level. We then performed in vitro capillary tube formation assay in SK-HEP-1 and HCC-LM3 cells, we found that TCM from the miR-182 group displayed more ability to promote the formation of capillary-like structures than the NC group, while the anti-miR-182 group decreased the level of capillary-like structures (Fig. 2c). These data indicate that miR-182 is overexpressed in HCC tumors and could increase the angiogenesis of HCC.

### RASA1 is the direct target of miR-182 and inhibits angiogenesis

**Fig. 3:**
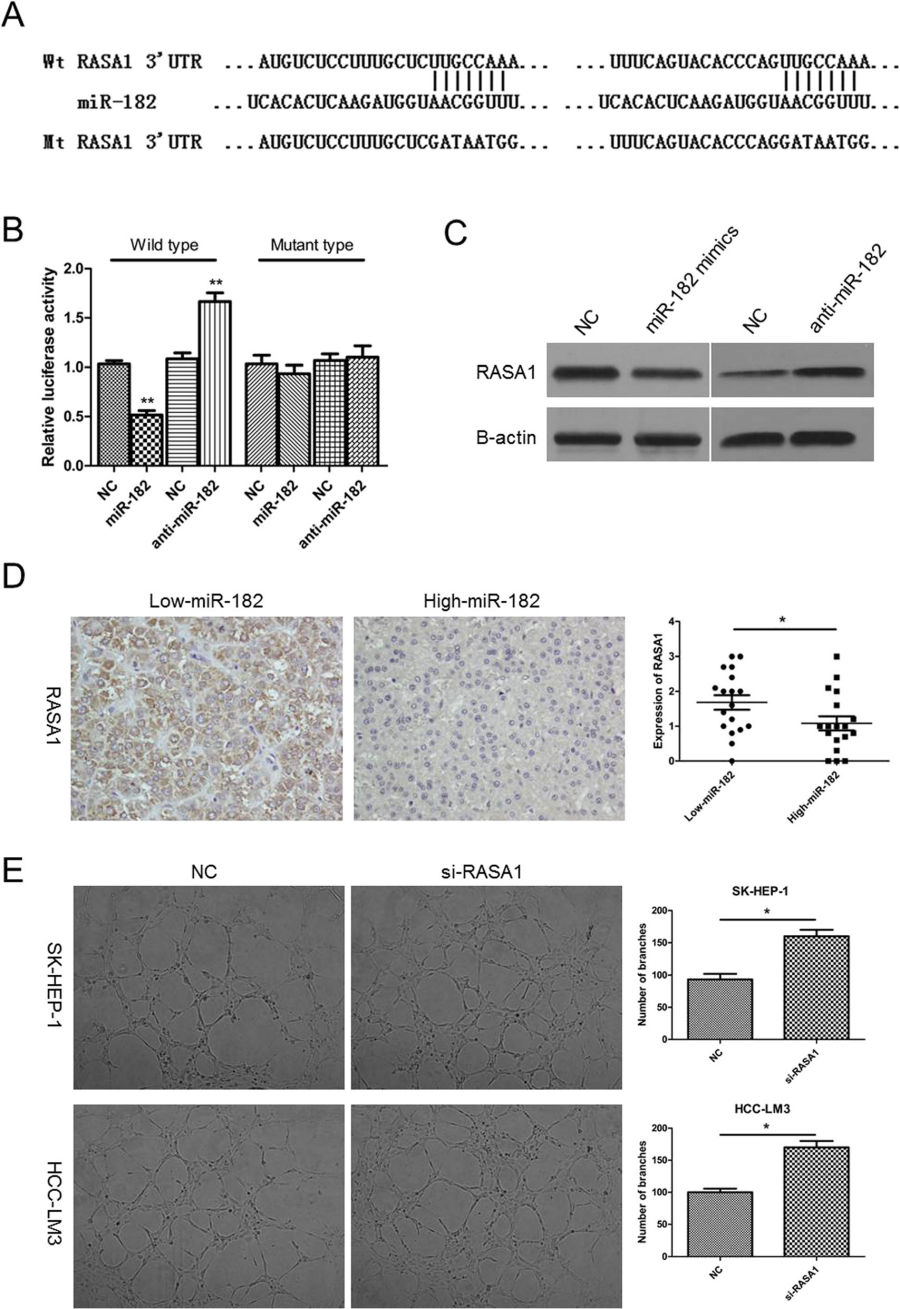
RASA1 was the direct target of miR-182 and the inhibition of RASA1 promoted angiogenesis. **a** miR-182 and its predicted binding sequence in the 3’UTR of RASA1. The mutant sequence was constructed by changing their complementary sites. **b** The SK-HEP-1 cells were co-transfected with miR-182 mimics or inhibitors or NC and 100 ng firefly luciferase reporter plasmid containing wild-type or mutant type 3’UTR of RASA1. After incubation for 48 h, the firefly luciferase activity of each sample was detected and normalized to the renilla luciferase activity. The data represent the mean ± SEM of triplicates. **c** The protein levels of RASA1 were examined by western blot after transfected with miR-182 mimics or inhibitors. **d** IHC assays were applied to explore the protein levels of RASA1 in 36 HCC tissues. The representative images were shown at the 400× magnification. The medium level of all 36 cases was chosen as the cut-off point for separating low-miR-182 (n = 18) and high-miR-182 (n = 18) groups. **e** Capillary tube formation assays was used to detect the angiogenesis effects of RASA1 suppression. The data represent the mean ± SEM of triplicates. (*P < 0.05, **P < 0.01)

It is well reported that the functions of miRNAs are mediated by their target genes. So we applied the database TargetScan and miRanda to search for the potential target genes of miR-182. Among the predicted genes, we focused on RASA1, because not only it was indicated by both databases (Fig. 3a), but also it has been reported to correlate with angiogenesis in a previous study [17]. The dual luciferase reporter results in SK-HEP-1 cells showed that the transfection of miR-182 mimics exerted repressive effects of luciferase activity of RASA1 3’UTR plasmid while the inhibition of miR-182 increased the luciferase activity (Fig. 3b). Moreover, the RT-PCR and western blot results in HCC cells lines indicated that overexpression of miR-182 reduced the mRNA level and protein level of RASA1 while the suppression of miR-182 revealed an opposite result (Fig. 3c and Additional file 1: Figure S1A). Based on the correlation in HCC cells, we then explore the level of RASA1 and the relationship between miR-182 and RASA1 in 36 HCC tissues. As shown in Fig. 3d and Additional file 1: Figure S1B, the expression of RASA1 was reduced in HCC tissues and the tissues with higher miR-182 levels tended to have the lower expression of RASA1. Furthermore, TCM from si-RASA1 group promoted the formation of capillary-like structure (Fig. 3e), which was consistent with miR-182 mimics transfection. Collectively, these findings imply that RASA1 is a direct target of miR-182 in both HCC cells and tissues.

### RASA1 is repressed by hypoxia and miR-182 mediates hypoxia-reduced RASA1 level

**Fig. 4:**
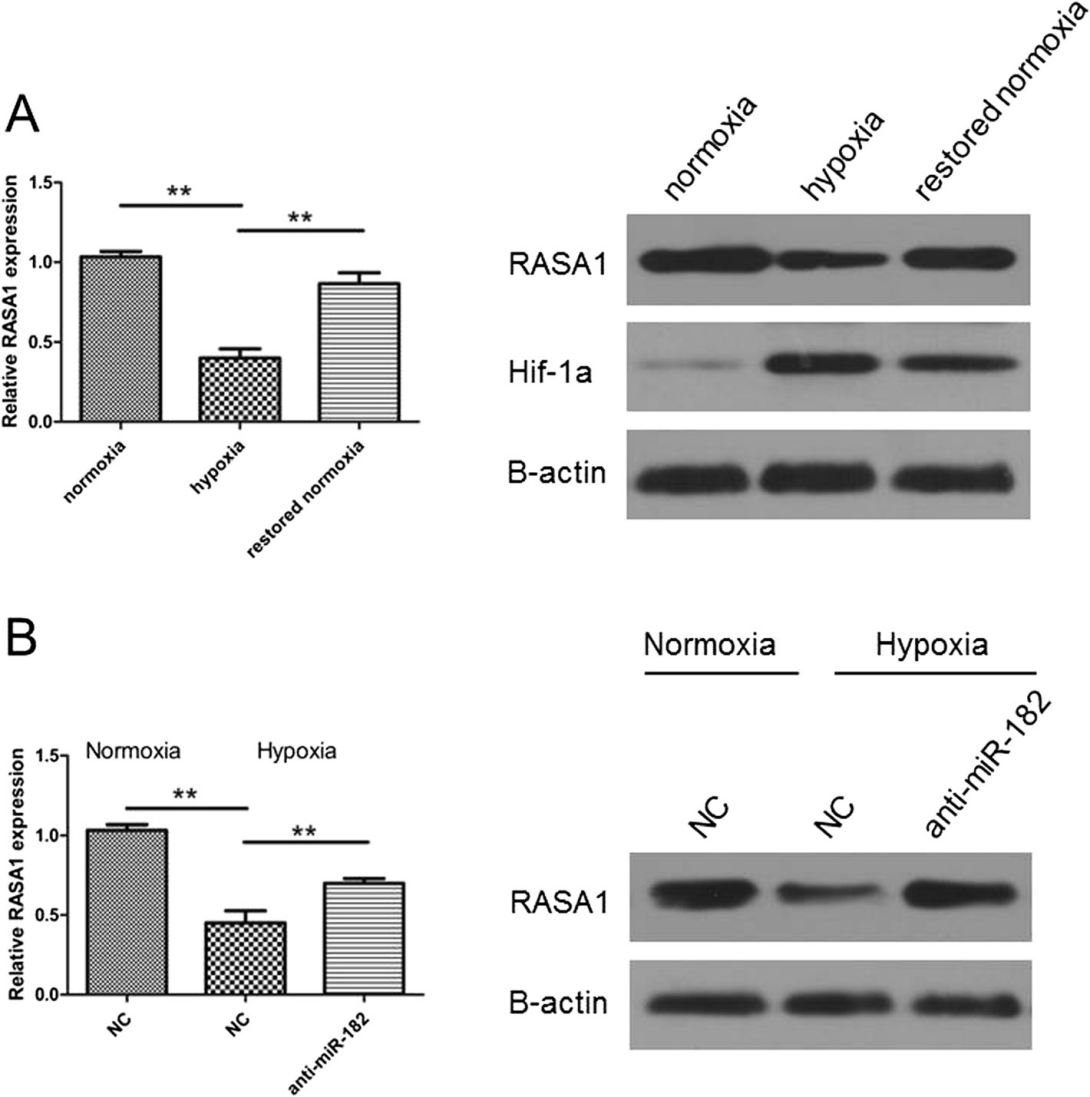
The level of RASA1 was reduced under hypoxia conditions and partly restored while transfected with anti-miR-182. **a** The mRNA and protein levels of RASA1 in SK-HEP-1 cells under normoxia or hypoxia or restored to normoxia were detected by RT-PCR and western blot. **b** SK-HEP-1 cells were transfected with miR-182 inhibitor or negative control in normoxic or hypoxic conditions. The mRNA and protein levels were detected. **a-b** The results indicate triplicates. (**P < 0.01)

To further determine the relationship between miR-182 and RASA1 under hypoxic conditions, we first explored the effects of hypoxia on RASA1. The results demonstrated that the mRNA and protein expression of RASA1 in HCC cells was significantly decreased when exposed to hypoxia (Fig. 4a). In addition, the RASA1 levels were significantly increased after the cells were restored to normoxic conditions (Fig. 4a). Given that RASA1 was the target of miR-182, we hypothesized that miR-182 might play a role in regulating hypoxia-reduced RASA1. To test this hypothesis, we transfected the SK-HEP-1 cells with miR-182 inhibitors. We found that the inhibition of miR-182 hindered the suppression effects of hypoxia on RASA1 expression (Fig. 4b). In conclusion, these results indicate that the reduction of RASA1 is mediated by up-regulation of miR-182 under hypoxic conditions.

## Conclusion

Increasing studies have proven that hypoxia is involved in promoting tumor progression and resistance to therapy [18]. In HCC, hypoxia has been reported to closely related to the proliferation, metastasis, angiogenesis and the outcome of patients [19–21]. Therefore, searching for novel hypoxia related markers might have potentially important clinical applications. A previous report has shown that the hypoxia-inducible miRNA, miR-210, augments the metastatic potential of HCC by targeting VMP1 [10]. Here, we sought to identify new hypoxia-induced miRNAs of HCC through microRNA microarray of hypoxic group and normoxic group in SK-HEP-1 cells. We selected the time point of 8 h, because the level of Hif-1a, which is the vital marker of hypoxia, was relatively higher than other groups. Among the differential miRNAs, miR-182 is a remarkable one with the fold change > 2 and P value < 0.01. Furthermore, upon restored to the normoxic conditions, the level of miR-182 significantly decreased. Till now, this is the first attempt to illuminate the dynamic change of miR-182 under hypoxia.

The importance of miR-182 in cancer has been reported in several studies. Previous researches have shown that miR-182 is up-regulated in prostate cancers [22], colorectal cancers [23]. Similarly, here we found that miR-182 was enhanced in most of the HCC tissues. Analysis between clinicopathological features and miR-182 expression revealed that the increase of miR-182 was significantly correlated with tumor size and portal invasion. These findings together indicate that miR-182 may acts as a valuable oncogene in HCC and further functional mechanism of miR-182 need to be explored to support its significance. A previous study in HCC reported that miR-182 regulates the metastasis activity by targeting MSPP1 [14]. But the potential role of miR-182 in other pathological progressions, such as angiogenesis, remains unclear.

Angiogenesis is vital for tumor growth and metastasis, which will accelerate the death of patients with cancer [24, 25]. Hence, identifying anti-angiogenesis targets is thought to be effective in treating cancers. Recently emerging studies have reported angiogenesis-related markers in HCC [26–28]. In the present study, we first reported that miR-182 promoted angiogenesis in HCC by applying HUVEC. Hypoxia is thought to be an important factor to promote angiogenesis, mainly by up-regulating VEGFA [29, 30]. Our results highlight another important molecule induced under hypoxia, which promotes angiogenesis, and might enhance the malignancy of tumors. Furthermore, during the investigation of the mechanism of miR-182 in HCC progression, we discovered that RASA1 was the direct target of miR-182 by the following evidence: miR-182 level and RASA1 expression are inversely correlated in both HCC cells and tissues; miR-182 directly binds to the 3’UTR regions of RASA1. RASA1, acting as the suppressor of RAS functions, is involved in the tumorigenicity of colorectal cancer [31] and gastric cancer [32]. Another research has revealed that loss of RASA1 promotes endothelium pathological angiogenesis [17]. Here we found that silencing of RASA1 could mimic the effects of miR-182 overexpression on angiogenesis. These results indicate that the increase of miR-182 promotes angiogenesis via down-regulating RASA1 in HCC.

In conclusion, we identified that miR-182 was induced in HCC cells under hypoxia and promoted angiogenesis by targeting RASA1. These findings facilitate our understanding of hypoxia related markers in HCC and the hypoxia/miR-182/RASA1 pathway might serve as a promising strategy for HCC therapy.

## Additional file

Additional file 1: Figure S1. The mRNA level of RASA1 in HCC cells transfected with miR-182 mimics or inhibitors and in HCC tissues. (A) miR-182 mimics increased while miR-182 inhibitors decreased the mRNA level of RASA1 in SK-HEP-1 cells. (B) The mRNA level of RASA1 were detected by RT-PCR in 36 HCC tissues. Compared to the non-tumor group, the tumor group showed a less level of RASA1.

## Abbreviations

HCC: Hepatocellular carcinoma
miRNAs: microRNAs
3’-UTR: 3’ un-translated regions
RNU6B: U6 small nuclear RNA
HUVECs: Human umbilical vein endothelial cells
