# Supplementary figures and images for: Hypoxia-inducible MiR-182 promotes angiogenesis by targeting RASA1 in hepatocellular carcinoma
Source: J Exp Clin Cancer Res. 2015 Jun 28;34(1):67. doi: 10.1186/s13046-015-0182-1 (PMC4493986; doi:10.1186/s13046-015-0182-1)

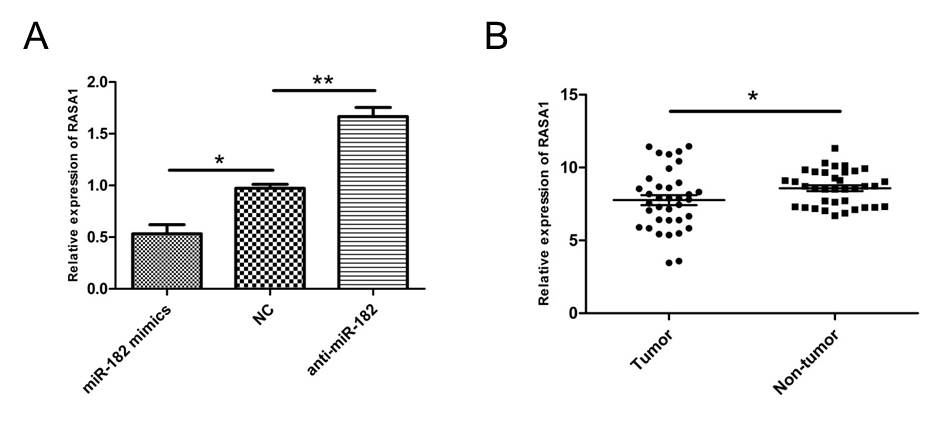

Supplement: Additional file 1: Figure S1. — The mRNA level of RASA1 in HCC cells transfected with miR-182 mimics or inhibitors and in HCC tissues. (A) miR-182 mimics increased while miR-182 inhibitors decreased the mRNA level of RASA1 in SK-HEP-1 cells. (B) The mRNA level of RASA1 were detected by RT-PCR in 36 HCC tissues. Compared to the non-tumor group, the tumor group showed a less level of RASA1. [file 13046_2015_182_MOESM1_ESM.jpg]
